# An Active Learning Framework for PFAS-Free Surfactant Discovery in Firefighting Foams

**DOI:** 10.1021/acsomega.6c07702

**Published:** 2026-09-30

**Authors:** Xin Wang, Hongwei Zhang, Xingtong Yu, Seokgyun Ham, Brian Lattimer, Rui Qiao

**Affiliations:** † 1757 Virginia Tech, Department of Mechanical Engineering, 635 Prices Fork Road, Blacksburg, Virginia 24061, United States; ‡ 26451 The Chinese University of Hong Kong, Department of Systems Engineering and Engineering Management, Ma Liu Shui, New Territories, 999077 Hong Kong, China

## Abstract

The growing concerns over the environmental and health impacts of per- and polyfluoroalkyl substances (PFAS) in firefighting foams have necessitated the development of efficient discovery frameworks for eco-friendly alternatives. In this work, we demonstrate an integrated pipeline leveraging active learning and coarse-grained (CG) molecular dynamics simulations to explore the design space of single-tail hydrocarbon surfactants for inhibiting fuel transport across interfaces. We design a targeted chemical space of 12,124 CG surfactants, represented by latent vectors learned from molecular graphs using a GNN-based autoencoder. Through four rounds of active learning, simulating only 2.5% of this space, we identify top-performing candidates with calculated interfacial transport resistance (*R*_int_) reaching 1641.5 s/m, representing a significant improvement over the commercial DTAB benchmark. Furthermore, we evaluate the effectiveness of constant and adaptive sampling strategies in Bayesian Optimization during the discovery process. Overall, this work establishes an active-learning-driven computational paradigm for accelerating the discovery of PFAS-free surfactants, providing both chemical design insights and an open-access data set to guide future experimental synthesis and validation. All data and source code used in this study are publicly available at https://github.com/elliewang21/Active_Learning.

## Graphical abstract

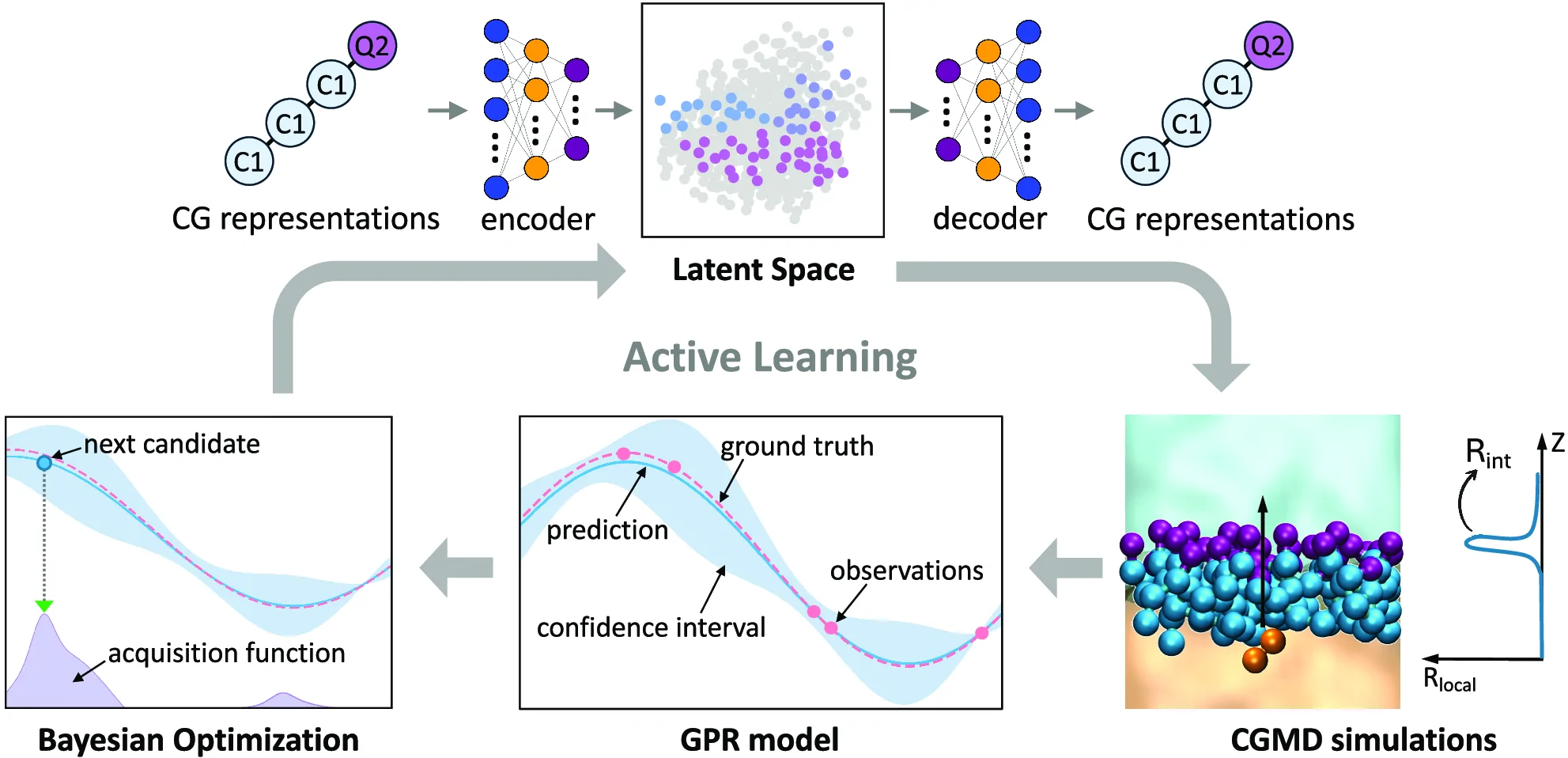

## 1 Introduction

Foams based on fluorinated surfactants, such as per- and polyfluoroalkyl substances (PFAS), have been widely used in firefighting foams for suppressing Class B liquid fuel fires.^1–4^ Despite their proven effectiveness, PFAS have been restricted globally due to their adverse effects on human health and persistence in the environment.^5^ In response, two primary research directions have emerged. First, extensive efforts have focused on developing effective methods for PFAS removal from contaminated sites.^6–10^ Second, the development of alternative surfactants to replace PFAS has gained momentum.^11,12^

The efficient development of effective PFAS alternatives for firefighting foams can benefit from a fundamental understanding of the fire suppression mechanisms of foams.^13^ Among the many roles that foams play in extinguishing liquid fuel fires, the significant suppression of fuel release from liquid fuels to the environment is widely considered an essential requirement.^14^ Fuel transport through firefighting foam is a multiphysics process that involves numerous substeps, such as the solubilization of fuel into the foam solution, transport of fuel in the lamellae separating bubbles, revaporization in gas bubbles, and redissolution in solution around bubbles. Many of these steps can proceed through multiple pathways, e.g., fuel transport in the aqueous lamellae can occur by diffusion and convection, and diffusion itself can occur through bulk water or through self-assembled nanostructure (e.g., micelles) dispersed within the water.

Surfactants play an important role in many of the above processes and thus affect the foam’s performance in controlling fuel release. Recently, the suppression of fuel transport across fuel-water interfaces by surfactants has been identified as a key factor in foam’s fire suppression efficiency.^15,16^ Designing surfactants to minimize such interfacial transport is thus a promising direction in developing PFAS alternatives.^17^ In principle, both the head and tail groups of surfactant molecules should be optimized during the design. However, recent molecular dynamics (MD) simulations of heptane transport across water-vapor interfaces featuring a sodium dodecyl sulfate (SDS) monolayer revealed that the interfacial transport resistance (*R*_int_) is dominated by the surfactant headgroup.^18^ Specifically, the fuel transport resistance resides primarily in the headgroup-solution region, while the contributions from the surfactant tail region are minor. The dominant role of the surfactant headgroup originates from the less favorable partition and more restricted diffusion of fuel molecules in the surfactant head-rich interfacial region than in the tail-rich interfacial region. These results suggest that strategic manipulation of the headgroup could guide efforts to identify promising surfactant candidates.

Exploring the vast chemical space of potential surfactants, even when focusing on variations of their head groups only, still poses substantial challenges due to the high computational cost of evaluating each candidate. Machine learning (ML) technologies, particularly active learning,^19^ have advanced as powerful tools for navigating such spaces efficiently.^20^ By iteratively selecting the most informative data points, active learning reduces the need for exhaustive sampling, significantly accelerating the screening process while conserving computational resources.^21^ In molecular design, active learning protocols combined with predictive models have shown promising results in uncovering novel molecules with desired properties.^22^ For example, Mohr et al.^23^ implemented an active learning framework to identify organic compounds with high selectivity for membranes, and Doan et al.^24^ demonstrated a 5-fold improvement in computational efficiency using Gaussian Process Regression (GPR) and Bayesian Optimization (BO). In addition, our previous work^25^ has shown that graph neural networks (GNN)-based models can effectively predict fundamental surfactant properties, such as critical micelle concentration (CMC) and surface tension, highlighting the promise of graph-based molecular representations for surfactant design.

In this work, we present an active learning framework combined with coarse-grained molecular dynamics (CGMD) simulations to enable efficient screening of surfactants with high interfacial transport resistance, *R*_int_ (see Figure 1). We focus on surfactants with hydrocarbon tails because of their more facile synthesis and lower cost.^26^ The key contribution of this work lies in applying and integrating established computational approaches into a data-efficient workflow for discovering PFAS-free surfactants, with *R*_int_ serving as a physics-based optimization target. In particular, the framework couples physics-based CGMD evaluation with uncertainty-aware active learning to efficiently navigate the surfactant design space without exhaustive simulation of every candidate. Our approach leverages a GNN-based autoencoder for molecular representation, GPR surrogate models, and BO to guide sampling within the chemical space. We employ coarse-grained (CG) representations of surfactants to streamline the chemical space by focusing on distinct chemical motifs, thereby enhancing computational efficiency. This framework allows us to rapidly explore a targeted space of 12,124 CG surfactants, filtered from an initial space of 2.8 million hydrocarbon surfactants. By simulating only 2.5% of this space, we identify high-performing candidates with high *R*_int_ values without relying on exhaustive CGMD screening. Additional contributions include the extraction of interpretable structure–property relationships from the sampled design space (i.e., how interfacial fuel transport resistance is related to surfactant structure), which can provide molecular-level design guidance and reusable data for future computational and experimental studies.

**Figure 1:**
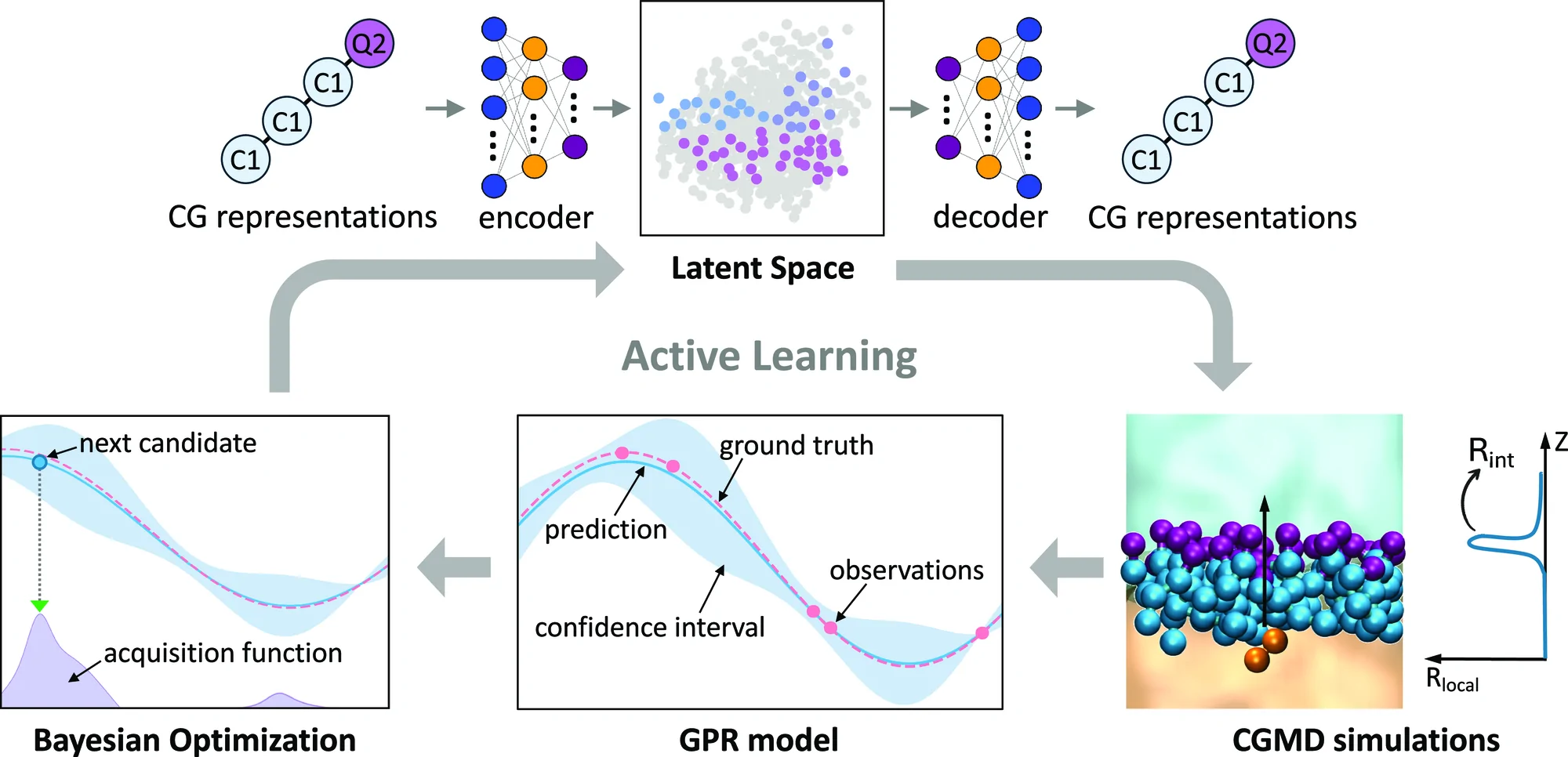
An active learning framework for discovering surfactant molecules with high resistance to fuel transport across oil–water interfaces. The framework combines CGMD simulations and Bayesian Optimization to efficiently explore the chemical space of candidate surfactants.

The rest of the manuscript is organized as follows: Section 2 details the methods, including the chemical space definition, CGMD simulations, and active learning framework; Section 3 presents the results of the discovery pipeline and discusses the resulting design insights; and Section 4 draws final conclusions.

## 2 Methods

### 2.1 Chemical Space of Surfactant Molecules

The objective of the chemical space design is to construct a surfactant library for the discovery task. We fixed the surfactant tail as a linear hydrocarbon tail and varied only the head groups in a linear configuration to maintain a manageable chemical space. The surfactant chain length significantly impacts foam performance;^14^ a 12-carbon (C12) tail strikes an optimal balance between interfacial properties and molecular size. Compared to C8 and C16 surfactants, the C12 surfactants ensure adequate hydrophobicity for interfacial stability while preserving solubility and manageable viscosity, making them promising for firefighting applications.

Surfactant molecules were constructed from chemical motifs presented using CG beads in the Martini 3 force field.^27^ In the Martini 3 force field, on average, four heavy atoms and their associated hydrogens are mapped into one CG bead, preserving the original volume and shape of the original chemical group. The coarse-grained description of molecules using the Martini 3 framework has demonstrated great success in applications ranging from self-assembly of peptides^28^ and small solute transport across membranes^29^ to membrane-protein interactions.^30^ Martini 3 has also been applied to micelle-forming surfactants; for example, Cabezudo et al. developed a Martini 3 model for dodecylphosphocholine (DPC) that reproduced micelle formation with aggregation numbers in good agreement with experimental values.^31^ The correspondence between CG beads and common chemical motifs (building blocks) has been tabulated.^27^ This building-block approach enables the efficient construction of CG representations of candidate surfactants using transferable Martini 3 bead types, while retaining the physicochemical interaction characteristics encoded by the force field.

In this study, surfactant tails are represented by a hydrophobic C1–C1–C1 bead sequence. Surfactant head groups are built from a head bead library composed of 28 unique beads, spanning neutral, negatively charged, and positively charged beads with a wide range of hydrophilicity. The bead types, charges, oil-to-water transfer free energies, and bead roles used in the enumeration are provided in Table S1. Our design of surfactant molecules’ chemical space followed a hierarchical approach. First, we constructed surfactant molecules by enumerating all possible real molecules corresponding to potential CG bead combinations and then applying screening methods to filter them. Next, the screened real molecules were mapped back to bead-based representations, further reducing the number of candidates. The specific implementation steps are shown in Figure 2.

**Figure 2:**
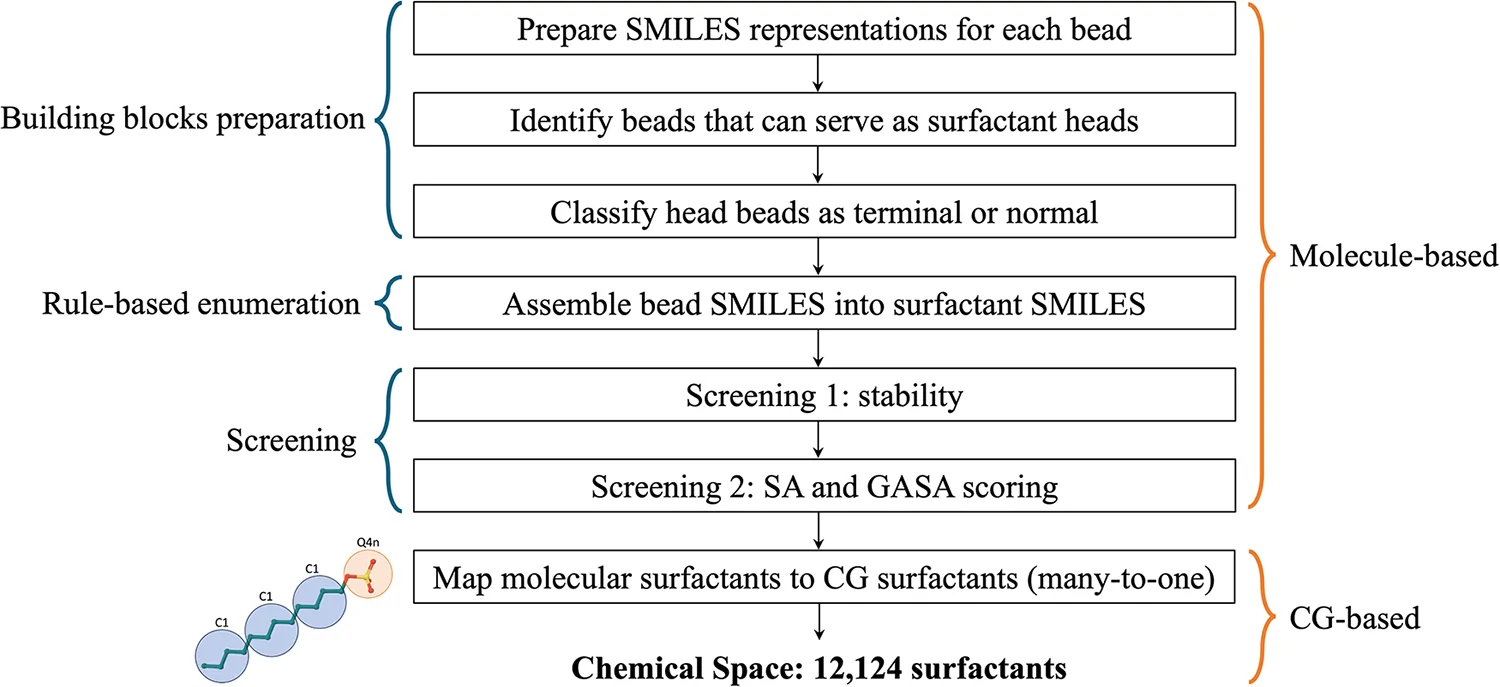
Workflow for constructing the chemical space of candidate surfactants.

Supporting Tables 24 and 25 of ref. 27 provide default Martini 3 bead assignments for common neutral molecules/fragments and ionic species, respectively. Building upon these assignments, we curated plausible 2D chemical topologies for each bead using standard organic chemistry textbooks and chemical databases (e.g., PubChem),^32–34^ from which we enumerated all possible structures and identified the maximum number of connectable sites based on normal atomic valence while accounting for common exceptions. CG beads were categorized into terminal-only beads, which can only appear at the terminal position of the surfactant head, and normal beads, which can appear at both internal/connectable and terminal positions. For both types of beads, we listed all possible SMILES formulas for different combinations of connectable sites. For normal beads, the two sites were as far apart as possible to maintain a linear molecular geometry. Isomers were also considered in this enumeration.

To prepare the bead-based real-molecule blocks for connection, we specified a set of connection rules. Surfactant molecules were then constructed sequentially from the tail (left) to the head (right), allowing direct connections for certain special cases involving beads with different charges. The tail was represented as a 12-carbon unit serving as a building block. Once the SMILES blocks were prepared, the SMILES string of the entire molecule could be generated directly by concatenating the corresponding blocks. Construction always began with the tail block and ended with the head blocks. To balance structural diversity, stability, and synthetic tractability, headgroup lengths were limited to one to four beads. Notably, normal beads could occupy any position within the headgroup, whereas terminal-only beads could appear only at the terminal position. After enumerating all possible combinations, the total number of potentially valid surfactant molecules was ∼2.8 million. We then applied two rounds of screening to reduce the size of the design space.

The initial screening criterion was the stability of the surfactant. Given an oil–water transfer Δ*G* of 51.6 kJ/mol^27^ for the tail, we conducted several MD simulations and observed that heads with a total oil–water transfer Δ*G* exceeding 40 kJ/mol can form a stable layering structure at the oil–water interface. Therefore, molecules with a total headgroup oil-water transfer free energy of less than 40 kJ/mol were excluded, reducing the candidate pool to ∼1.9 million.

Further screening was conducted using two scoring functions to ensure chemical viability. The Synthetic Accessibility (SA) scoring function evaluates synthesizability based on molecular structure,^35^ where a threshold of SA < 3 was applied to exclude structurally complex candidates. Additionally, a pretrained GASA model assessed synthesizability based on the predicted number of reaction steps for synthesis,^36^ where 0 represents 1–3 reaction steps and 1 represents >10 steps. A GASA < 0.5 filter was employed. Combining these two functions greatly narrowed the candidate pool to 23,744 potentially synthesizable molecules.

The 23,744 molecular candidates that passed the screening criteria were then mapped to their corresponding Martini 3 CG representations. During the initial molecular-space construction, each SMILES-based candidate was associated with its corresponding ordered CG bead sequence. Because the Martini representation is inherently many-to-one, different molecular structures can map to the same ordered CG bead sequence. Candidates with identical bead sequences were therefore treated as a single CG surfactant for subsequent CGMD simulations and active-learning calculations. After removing these duplicate CG representations, the final design space consisted of 12,124 unique CG surfactants.

### 2.2 Coarse-Grained Molecular Dynamics (CGMD) Simulations

#### 2.2.1 Simulation System and Molecular Models

The system used to evaluate the interfacial transport resistance (*R*_int_) of octane molecules across oil–water interfaces covered with surfactants features a water slab, two octane slabs, and two surfactant monolayers accumulating at the oil–water interfaces (see Figure 3). Here, octane is selected as the model fuel molecule because it is a chemically simple, representative midchain hydrocarbon of common liquid fuels,^37^ and robust coarse-grained parametrizations are available in Martini 3.^27^ The upper surfactant monolayer was fixed to prevent the collective movement of the system in the *z*-direction. The lower monolayer, water, and octane are mobile. The water layer includes the sodium ions or chloride ions to neutralize the system when surfactants are charged. The initial simulation system typically measures 4.47 × 4.47 × 15.1 nm^3^. The periodic boundary condition applies to all directions. The thicknesses of the water slab and octane slab are ∼6.8 nm, and ∼5.0 nm, respectively, allowing the formation of two bulk-like regions in their middle.

**Figure 3:**
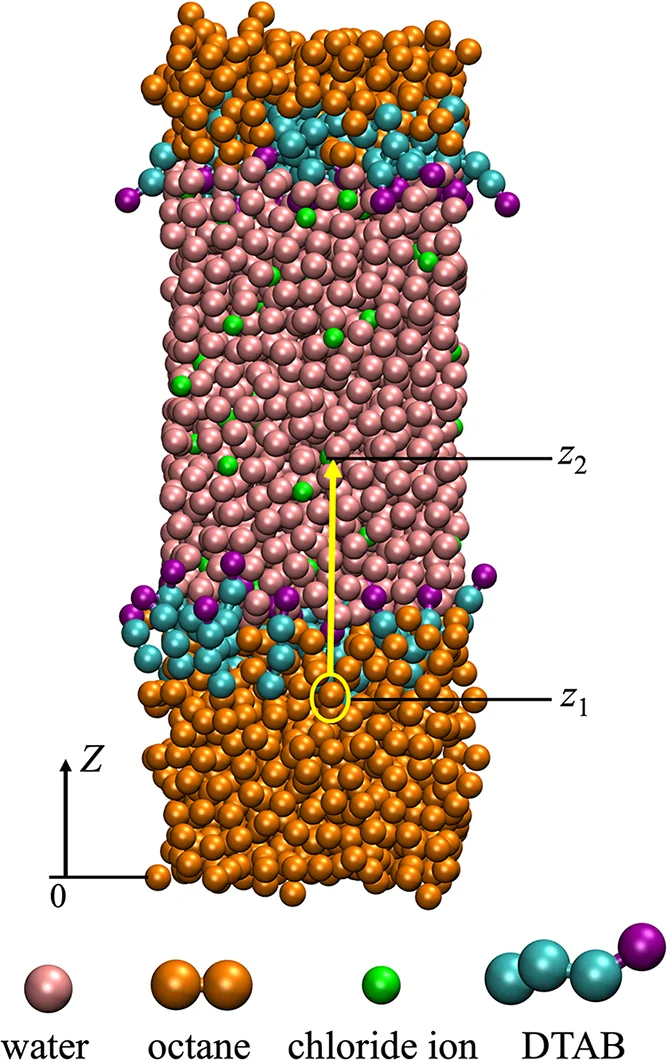
A snapshot of the MD system used to compute the *R*_int_ of octane molecules across an octane–water interface covered by a DTAB surfactant monolayer. *z*_1_ and *z*_2_ denote the region where the transport of the target oil molecule (circled by the yellow line) is studied.

All molecules were modeled using the Martini 3 force field.^27^ Due to the structural simplicity of our target surfactants, we employed the default bond length of 0.47 nm with a force constant of 3800 kJ/mol/nm^2^, as well as the angle of 180° with a force constant of 35 kJ/mol to construct the surfactants. The bonded parameters were adopted from the saturated hydrocarbon-chain parameters in the Martini 3 phospholipid topology, which can be accessed in the official Martini 3 Web site.^27^ The default W bead, a nonpolarizable water model, was adopted to represent the water molecules.

#### 2.2.2 Simulation Protocol and Methods

To determine the *R*_int_ of octane molecules, we first identified the maximum surface density of surfactants at the oil–water interfaces because oil–water interfaces were typically saturated with surfactants in foam solutions. Using the systems with the identified maximum surface density, we then conducted umbrella sampling simulations to compute the potential of mean force (PMF­(*z*)) and the local diffusion coefficient (*D*(*z*)) of oil molecules across oil–water interfaces, from which *R*_int_ was computed.

##### 2.2.2.1 Determination of Maximum Surface Density of Interfacial Surfactants

We first built a series of systems by packing two monolayers of surfactants. Each monolayer of surfactants was constructed with varying numbers of surfactants, ranging from 40 to 112, corresponding to a surface density σ of 1.0–2.8 nm^–2^ with an interval of 0.2 nm^–2^. The CG water and octane molecules were then packed using the Packmol code.^38^ For each system, an energy minimization run was conducted using the steepest descent algorithm until the maximal force was below 100 kJmol^–1^ nm^–1^. This was followed by a 5 ns simulation in the canonical (NVT) ensemble at a temperature of 300 K. Then, a semi-isotropic NPT equilibrium simulation was performed at 1 bar for another 100 ns. After these simulations, the number density of surfactants’ head in each system was extracted from the last 80 ns of the NPT run. We then compared the surface headgroup density profiles in systems with various surfactant surface densities. The maximum surfactant surface density was defined as the highest surface density at which sharply defined headgroup density profiles were observed and the number density of head groups in the bulk oil/water regions remained negligible (<0.01 nm^–3^).

##### 2.2.2.2 Calculations of PMF­(*z*) and *D*(*z*) Using Umbrella Sampling

Before the umbrella sampling run, an NVT simulation of 40 ns was performed to equilibrate the system with the highest surface density of surfactants, where the upper surfactant monolayer was fixed to avoid the movement of the whole system in the *z*-direction. Then, the center of mass (COM) of an octane molecule was pulled toward the water phase from *z*_1_ = 4.0 nm to *z*_2_ = 7.9 nm by a harmonic spring with a pulling speed of 0.0001 nm/ps and a force constant of 2000 kJmol^–1^ nm^–2^. A total of 40 configurations corresponding to octane positions of *z* = 4.0–7.9 nm with an interval of 0.1 nm were saved. Then, another 1 μs NVT run was performed for each of the 40 systems. In these simulations, the COM of the octane molecule was constrained in the *z*–direction using a harmonic spring with a force constant of 1000 kJmol^–1^nm^–2^. PMF­(*z*) was calculated using the weighted histogram analysis (WHAM) algorithm. The local diffusion coefficient *D*(*z*) near the interfaces was determined using the position autocorrelation function (ACF)^39^

1 $$D\left(z=\left\langle z\right\rangle \right)=\frac{\mathrm{var}\left(z\right)}{\int_{0}^{\infty }\mathrm{ACF}\left(t\right)dt}$$

where var­(*z*) is the variance of the restrained octane molecule’s *z* positions and ACF­(*t*) is its position autocorrelation function. To improve the accuracy of *D*(*z*), each of the 40 trajectories, where the COM of the octane molecule was constrained at different *z*–positions for 1 μs, was divided into 20 short segments. From each subtrajectory, ACF­(*t*) was extracted. The local diffusion coefficient was then calculated for each subtrajectory and then averaged over all subtrajectories. Given that *t*_*c*_ is the characteristic decay time when ACF­(*t*) first falls below 0.05, for ACF­(*t*) with *t*_*c*_ ≤ 10 ps, ACF­(*t*) was integrated numerically up to 200 ps. For ACF­(*t*) with *t*_*c*_ > 10 ps, the integral was calculated as a sum of the numerical integration of ACF­(*t*) for *t* < *t*_*c*_ and an analytical integration of a fitted exponential decay function for *t_c_* < *t* < ∞, where the exponential decay function was fitted using the ACF­(*t*) within 0.2*t*_*c*_ ∼ *t*_*c*_.^18^

##### 2.2.2.3 Determination of *R*_int_

The calculation of *R*_int_ from the PMF and local diffusion coefficient follows the methodology established in the previous work.^18^ The local resistivity *R*(*z*) at different *z*–positions can be calculated using the inhomogeneous solubility-diffusion theory as^40^

2 $$R\left(z\right)=\frac{\exp\left(\frac{\mathrm{PMF}\left(z\right)}{k_{B}T}\right)}{D\left(z\right)}$$

where PMF­(*z*) and *D*(*z*) are the potential of mean force and local diffusion coefficient of the octane molecule across the oil–water interfaces, respectively. Integrating the local resistivity *R*(*z*) along *z* between *z*_1_ and *z*_2_ leads to the total transport resistance *R*_tot_ in the space between *z*_1_ and *z*_2_. To compute the resistance contributed by the surfactant monolayer only, we defined the *R*_int_ by subtracting the resistance contributed by a water layer from the total resistance

3 $$R_{\mathrm{int}}=R_{\mathrm{tot}}-R_{b}$$

4 $$R_{b}=w\cdot R\left(z_{2}\right)$$

where *R*_b_ is the resistance for the octane transport through a bulk water slab of thickness *w* = *z*_2_ – *z*_*g*_. *z*_*g*_ represents the position of the Gibbs dividing surface, defined as the *z*-coordinate where the number density of water molecules is equal to 50% of the number density of water molecules in the bulk region.

All simulations were performed using the GROMACS code (version 2021.3). A time step of 20 fs was employed. LINCS algorithm was adopted to enforce constraints. The temperature of all mobile molecules was maintained at 300 K using a velocity-rescaling thermostat with a time constant of 1.0 ps. In NPT runs, the pressure was controlled by Parrinello–Rahman method for semi-isotropic volume scaling, with a time constant of 12 ps and isothermal compressibility of 3 × 10^–4^ bar^–1^ in the *z*-direction. A cutoff of 1.1 nm was used to compute the Lennard-Jones potentials, and the potential shift Verlet was adopted as a Lennard-Jones potential modifier. Electrostatic interactions were treated using the reaction-field method with a real-space cutoff of 1.1 nm and a screening constant of 15.

### 2.3 Active Learning Framework

Through CGMD simulations, the interfacial transport resistance, *R*_int_, imposed by the surfactant monolayer at oil–water interfaces can be quantified for each surfactant candidate. However, since each simulation requires significant computational resources, it is imperative to navigate the surfactant design space and identify high-performing surfactants using a minimal number of evaluations. To achieve this, we employed an active learning framework centered on Bayesian Optimization (BO), a robust approach for global optimization of black-box functions.^41^ By incorporating prior knowledge of surfactant performance, BO balances exploration (sampling uncertain regions) and exploitation (sampling promising regions). In our framework, BO utilizes a probabilistic surrogate model to determine the distribution of the target variable, which is then used to compute a posterior-based acquisition function for candidate selection.

To capture both bead-specific and topological information, we represented surfactant molecules as molecular graphs. Node features included bead type and physicochemical labels, such as hydrogen-bonding, charge-related, self-interaction, and size labels. Molecule-level features included net charge and free energy. Edge features characterized connectivity via Lennard-Jones potential parameters, σ (the distance at which the particle–particle potential is zero) and ϵ (the depth of the potential well). Detailed definitions and ranges of these numerical and categorical features are provided in Table S2. To address the challenges of comparing molecules with varying structures and constructing probability distributions in high-dimensional matrix forms, we trained a GNN-based autoencoder to map these molecular graphs into a continuous 16-dimensional latent space $R^{16}$ .

The graph features were first processed through a message-passing neural network (MPNN).^42^ The resulting graph-level information was passed to a fully connected neural network to generate the 16-dimensional latent vector, **x**, which serves as the numerical representation of a CG surfactant. During training, a decoder was used to reconstruct the original graph representations from the latent vectors. The training phase was considered complete once 100% reconstruction accuracy was achieved. The resulting 16-dimensional latent vectors for all CG surfactants were then retained and used for subsequent K-means sampling, GPR-based Bayesian optimization, and latent-space analysis. The detailed architecture, loss function, and training procedure of the GNN-based autoencoder are provided in the Supporting Information.

In the latent vector space, initial sampling was performed using *K*-means clustering. The 12,124 molecules were partitioned into 100 clusters based on Euclidean distance, and the molecule closest to each centroid was selected for the initial set. The *R*_int_ values for these 100 representative molecules were calculated via CGMD simulations. These labeled data points were used to train a Gaussian Process Regression (GPR) model, which served as our probabilistic surrogate model. Specifically, the GPR model took the 16-dimensional latent vector **x** as input and produced a one-dimensional output, *f*(**x**), representing the predicted transport resistance, using a combination of radial basis function (RBF) and linear kernels.

Following the initial training, BO was employed to explore the unsampled design space. The model used the mean and variance predictions from the GPR to select new candidates through the Expected Improvement (EI) acquisition function. EI was chosen for its proven ability to balance exploration and exploitation across various optimization benchmarks.^43^ The EI function is defined as^43^

5 $$\mathrm{EI}=\left(\mu \left(x\right)-R_{int}^{*}-\xi \right)\Phi \left(\frac{\mu \left(x\right)-R_{int}^{*}-\xi }{\sigma \left(x\right)}\right)+\sigma \left(x\right)\phi \left(\frac{\mu \left(x\right)-R_{int}^{*}-\xi }{\sigma \left(x\right)}\right)$$

where μ­(**x**) and σ­(**x**) represent the mean and standard deviation of the predicted *R*_int_ for an unsampled point **x**, while *R*_int_^*^ denotes the maximum *R*_int_ value observed in the sampled set. The functions Φ and ϕ correspond to the cumulative distribution function (CDF) and probability density function (PDF) of the standard normal distribution, respectively. The parameter ξ regulates the trade-off between exploitation (sampling regions with favorable predicted values) and exploration (sampling regions with high uncertainty); a higher ξ value encourages broader exploration of the design space, whereas a lower value promotes local refinement.^44^ Both fixed and iteration-dependent choices of this parameter have been considered in previous Bayesian optimization studies. Kushner proposed using a relatively large exploration parameter initially and decreasing it as optimization progresses, while Lizotte later reported that such a decreasing schedule did not consistently outperform a constant value across benchmark problems.^44,45^ These alternative choices have also been discussed in later Bayesian optimization studies.^46^ In this study, we evaluated two distinct strategies for the ξ parameter in the discovery pipeline:

(a) **Constant sampling:** A fixed ξ = 0.01 was used to prioritize efficient exploitation throughout the process.
(b) **Adaptive sampling:** An initial ξ = 0.5 was used for the first two rounds to emphasize broader exploration, followed by a reduction to 0.01 in subsequent rounds to facilitate convergence on high-performance candidates.

In each iteration of the active learning loop, a batch of 50 candidates with the highest EI scores was selected for evaluation via CGMD simulations. The GPR model was then retrained using the newly acquired simulation data to update the *R*_int_ predictions for the remaining unsampled candidates. This iterative process continued until the discovery of improved candidates plateaued over several consecutive rounds.

### 2.4 Software

All CGMD simulations were performed using GROMACS, and initial molecular configurations were generated using Packmol. Chemical-space construction, data processing, molecular representation learning, Bayesian optimization, and subsequent analyses were implemented in Python. Major Python packages included Pandas and NumPy for data processing; PyTorch and PyTorch Geometric for the graph-based autoencoder; GPyTorch for Gaussian Process Regression; scikit-learn and SciPy for clustering, dimensionality reduction, and data preprocessing; and Matplotlib for data visualization.

## 3 Results and Discussion

### 3.1 CGMD Case Study

We used a representative CG surfactant, modeled by a bead combination of C1–C1–C1–SQ2p, to illustrate the process of calculating the interfacial resistance (*R*_int_). This CG surfactant is a suitable model for representing a commonly used commercial surfactant, dodecyltrimethylammonium bromide (DTAB). To determine the maximum surface density σ_max_, we varied the number of DTAB at the interface in a series of simulations, and then extracted the number density profiles of DTAB heads (see Figure 4a). The surface density (σ) of 1.60 nm^–2^ was identified as the maximum surface density (σ_max_) for DTAB surfactants since it has a prominent peak in the DTAB head density profiles with a stable interfacial structure.

**Figure 4:**
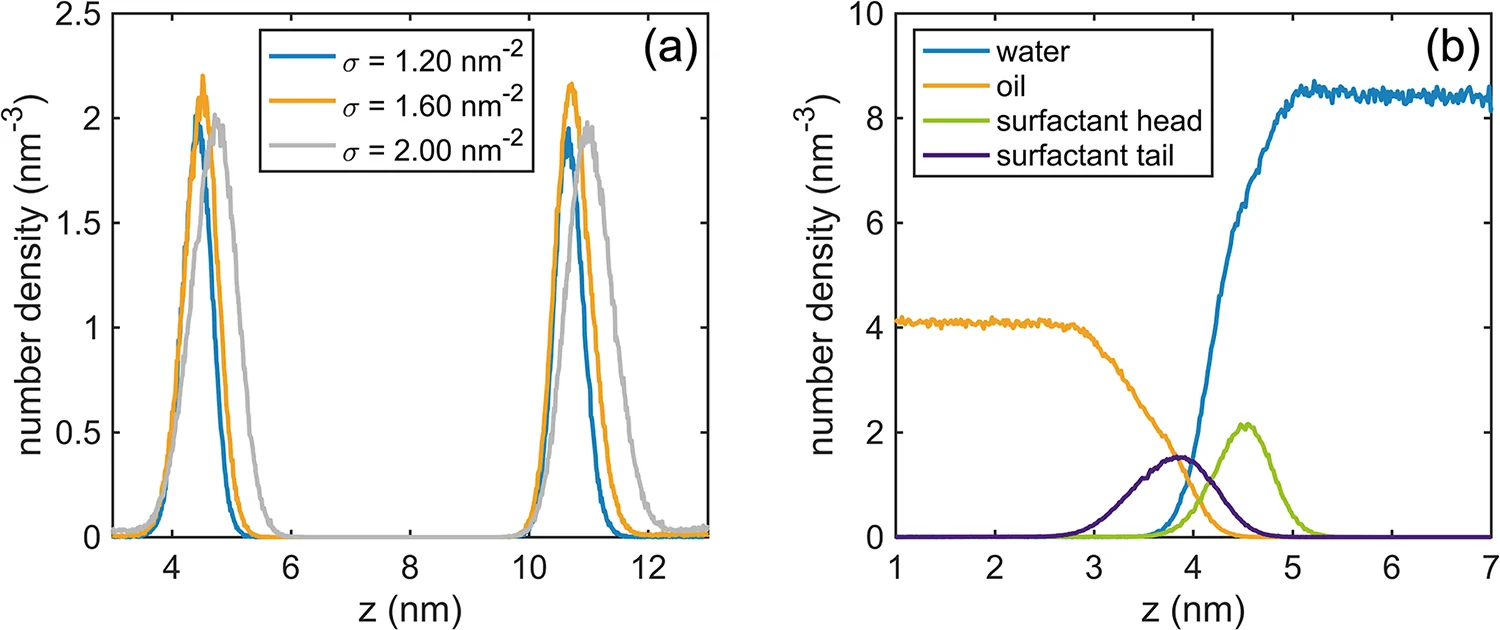
Number density profiles of (a) DTAB’s head groups with its surface density of 1.20 nm^–2^, 1.60 nm^–2^, and 2.00 nm^–2^, and (b) water, octane and DTAB surfactants near the interface with σ_max_ = 1.6 nm^–2^.

Figure 4b presents the number density profiles of DTAB, water, and octane near the oil–water interface at the maximum surface density σ_max_ = 1.60 nm^–2^. The number density of water and octane remains unchanged at *z* < 3 nm and *z* > 5 nm, respectively, signifying the formation of bulk water and bulk octane. The DTAB surfactants are self-assembled at the oil–water interface with their heads facing the water phase and tails oriented toward the oil phase. Since the interface between surfactant head and water, which is measured less than 1 nm in thickness, dominates the total transport resistance for fuel transport across the interface, as elucidated in our previous work,^18^ we focused our analysis on the PMF­(*z*) and *D*(*z*) within the region *z*_1_ = 4.0 nm to *z*_2_ = 7.9 nm, which is wide enough to cover those longer surfactants with up to 4 head beads.

After performing the umbrella sampling simulations, we obtained the PMF­(*z*) and *D*(*z*) of an octane molecule across the oil–water interface laden with DTAB surfactants, as shown in Figure 5a and 5b, using the methodologies outlined earlier. A clear PMF peak and low local diffusion coefficients are observed at *z* ≈ 5.2 nm, corresponding to the interface between the surfactant heads and water. This region of high PMF and slow octane diffusion therefore exhibits high local fuel transport resistivity (see Figure 5c). By integrating the local resistivity over *z* and subtracting the resistance contributed by the water (*R*_b_), we finally obtained the *R*_int_ imposed by DTAB on octane transport as 5.9 s/m, which is equivalent to the resistance of a water slab with a thickness of 6.9 nm. For the physical mechanisms behind the variation of the local resistivity across the surfactant-laden water interfaces, we refer interested readers to our previous study of heptane transport across SDS-laden water interfaces.^18^

**Figure 5:**
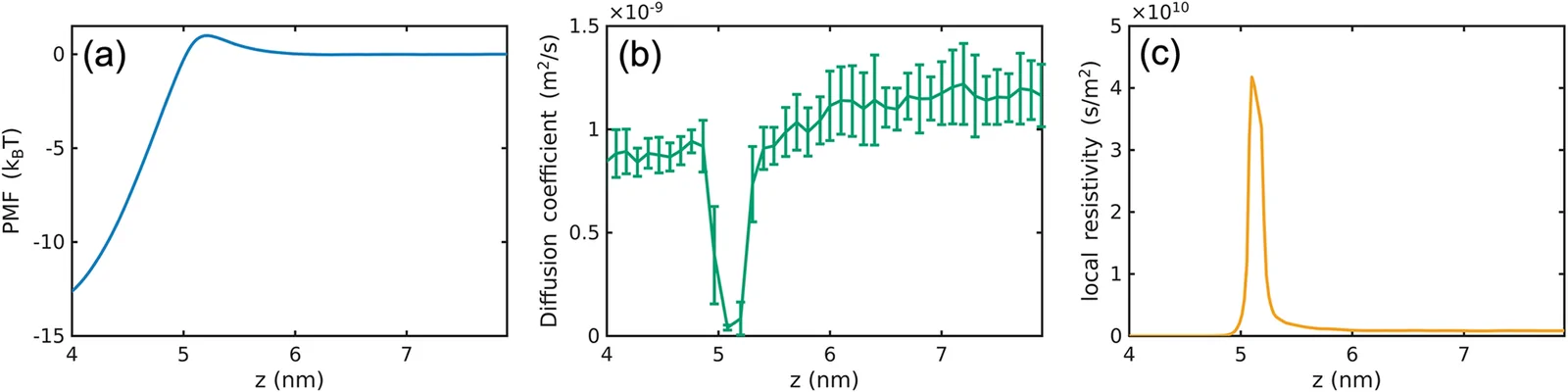
Potential of mean force (PMF) profile (a), local diffusion coefficient profile (b), and local transport resistivity (c) of an octane molecule across an oil–water interface covered by the self-assembled DTAB monolayer.

### 3.2 Surfactant Discovery by Active Learning

With the workflow described in Figure 1, four rounds of active learning-guided screening were performed following the initial sampling of 100 surfactant molecules. Two distinct sampling strategies were employed separately: adaptive and constant sampling, depending on whether the trade-off parameter (ξ) was held fixed or adapted. In each round, 50 additional molecules were selected for CGMD simulations guided by acquisition functions. In total, 300 molecules were tested for each sampling strategy. The evolution of top-performing candidates across rounds is depicted in Figure 6.

**Figure 6:**
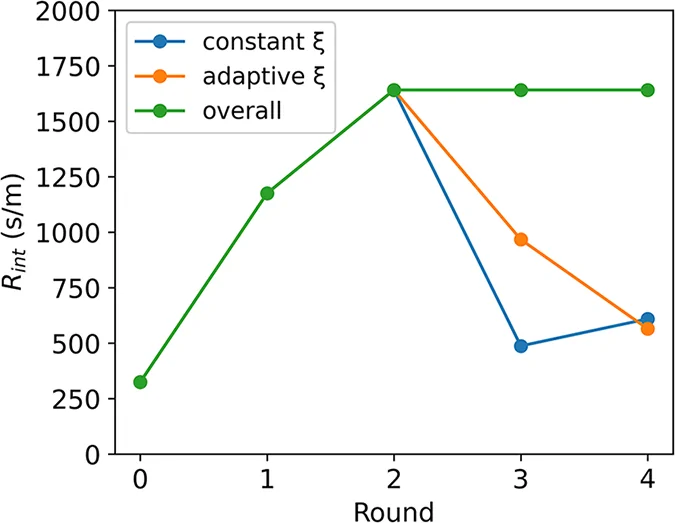
*R*_*int*_ results of the best performer in each round of active learning.

#### 3.2.1 Evolution Over Each Round

In the initial sampling round (round 0), without leveraging posterior information or active learning, the best *R*_int_ observed was below 350 s/m. Upon incorporating posterior knowledge and the acquisition function, the *R*_int_ increased to 1175.7 s/m in round 1, and further improved to 1641.5 s/m in round 2. Peak performance in this discovery task was achieved in round 2. Following this, both sampling strategies showed a decline in subsequent rounds, indicating that the top-performing candidates in this design space had already been identified. Notably, the top performers up to round 2 were identical across both strategies, highlighting the robustness of the active learning framework.

Although the best round-wise *R*_int_ values decreased after round 2 for both sampling strategies, the adaptive sampling appeared to outperform the constant sampling strategy, especially regarding the round-wise optimal candidate selection. To better compare the two sampling strategies, the *R*_int_ sampled in each round by two strategies are shown in Figure 7 where the overlapping and unique selection of each sampling strategy are presented separately. The adaptive strategy used a large trade-off factor ξ = 0.5 to penalize the exploitation in the first two rounds and then reduced ξ to 0.01 for the following rounds. In contrast, the constant strategy used ξ = 0.01 throughout all rounds.

**Figure 7:**
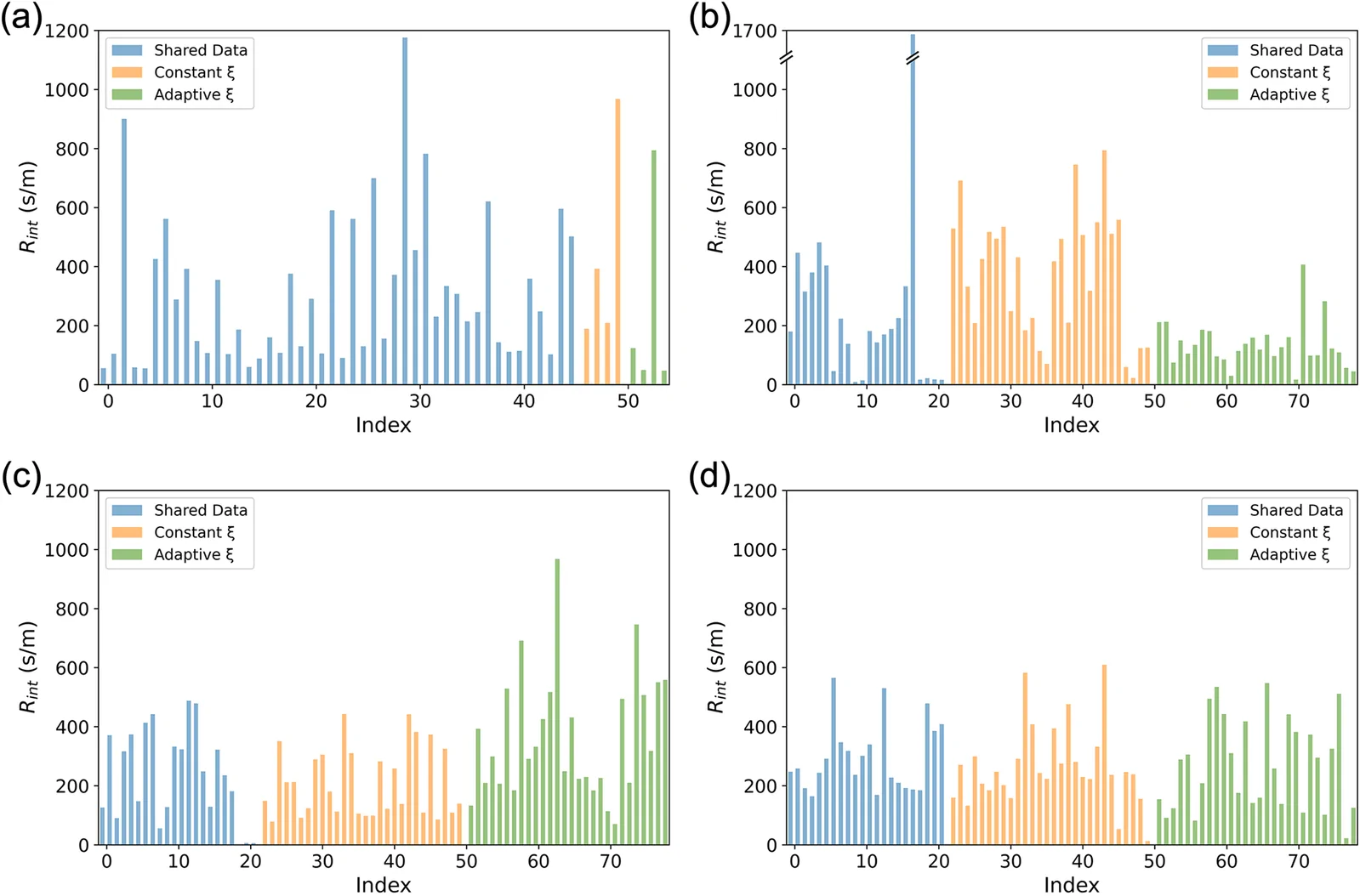
*R*_int_ selected in (a) round 1, (b) round 2, (c) round 3, (d) round 4 of the active learning. The *x*-axis is the number of unrepeated candidates selected by two sampling strategies. The candidates in blue are the overlap of the two strategies’ selections. The candidates in green (yellow) are the unique selections of the adaptive (constant) sampling strategy.

In round 1, in the absence of enough posterior information (the molecules were selected based only on clustering of the molecular information), the exploration term, namely, uncertainty in eq 5, is high over the majority of the sampling space. The two strategies tended to give the same suggestions when selecting the new candidates to be tested with only four unique selections, as the different trade-off factors control only the magnitude of the exploitation term, which is much smaller than the exploration term. In round 2, we noticed the overlapping selection between the two strategies shrank notably because the adequate probability distribution-based sampling greatly reduced the uncertainty. The appearance of the candidate with overall peak performance in the overlapping selections can be attributed to its significantly high *R*_int_ value, making it less dependent on the adjusted exploitation term. Comparing the performance of the unique candidates, the constant strategy outperformed the adaptive strategy with an overall higher *R*_int_. This can be traced back to the penalization of the exploitation in the adaptive strategy, which encouraged the exploration of the uncertain region instead of sampling the promising candidate based on the current posterior information.

In round 3, the adaptive strategy began to outperform the constant strategy, in contrast to round 2. Starting from this round, the trade-off factors were the same for the two strategies, so exploitation was no longer penalized. Given that the adaptive strategy has explored more uncertain regions, its surrogate GPR model is more accurate (*R* ^2^ = 0.96) than that of the constant strategy (*R* ^2^ = 0.94) and gives better searching advice. Comparing the *R*_int_ of the adaptively sampled candidates in round 3 to the constantly sampled candidates in round 2, it was found that the former exhibited much higher overall values, suggesting the advantage of using adaptive sampling over the constant sampling strategy. In round 4, the constant sampling strategy had explored more uncertain regions compared to the last round, so it obtained a similar performance to the adaptive strategy. However, if the sampling space is more complicated than the one in this work, the adaptive sampling will likely outperform the constant sampling for more rounds.

#### 3.2.2 Visualization of Sampling

Based on the discussion above, the key to the active learning sampling process was to balance exploitation and exploration within the 16-dimensional latent space. Unlike the original chemical space, the embedded space was designed to extract and retain the physical and chemical properties of the molecular representations in a compact manner. As an example, Figure 8(a) demonstrates the clustering of molecules with varying head lengths where the 16 dimensions were reduced to 2 dimensions, namely principal component 1 (PC1) and 2 (PC2), through the principal component analysis (PCA).

**Figure 8:**
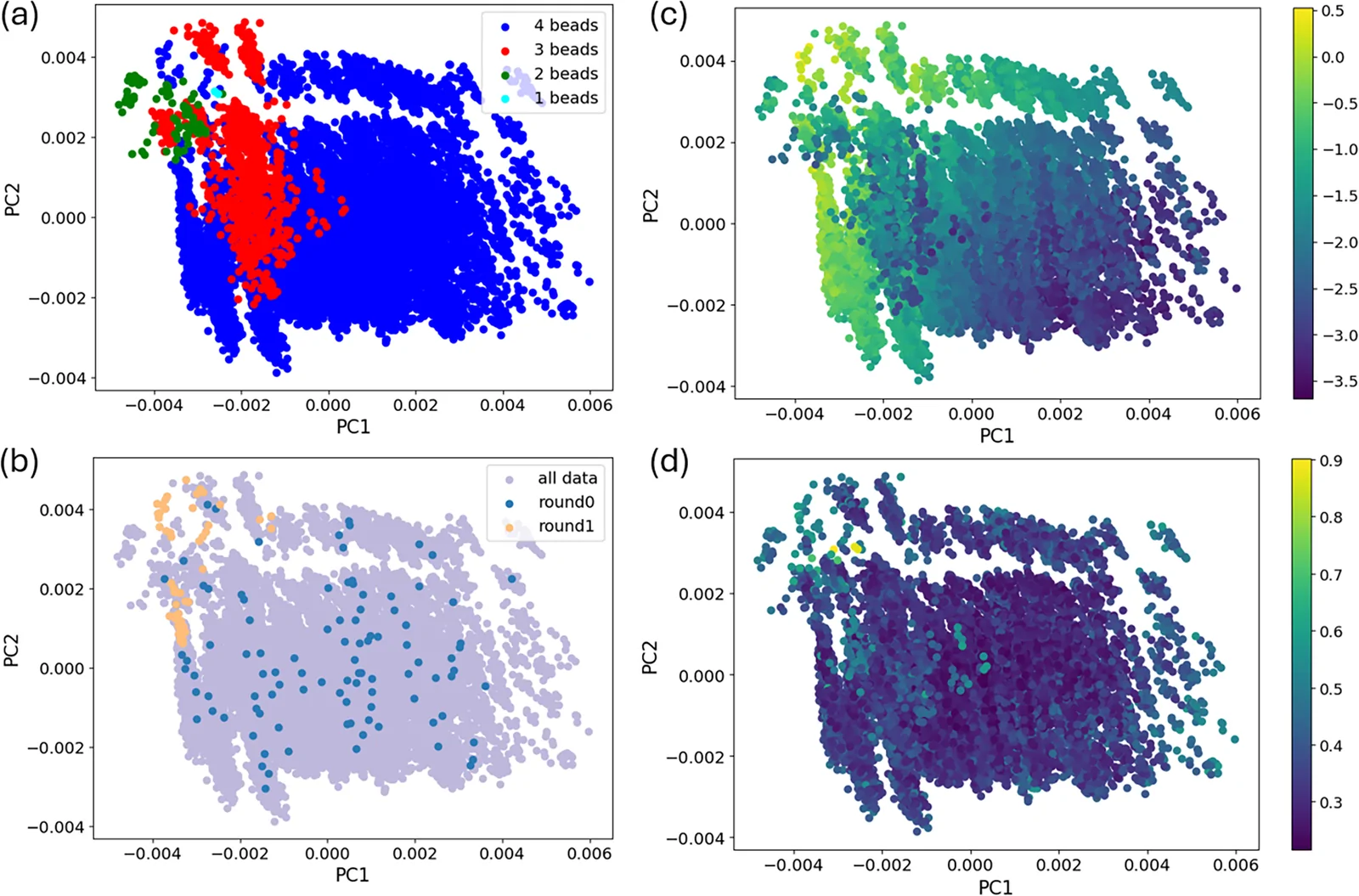
(a) The distributions of the molecule candidates with different head lengths projected in a two-dimensional space after PCA. (b) The spatial distributions of the sampled candidates in the initial sampling and the round 1 sampling. In the adaptive sampling strategy, (c) the exploitation term of each candidate in the round 1 selection in the space after PCA. (d) the exploration term of each candidate in the round 1 selection in the space after PCA.

Here, we used the sampling process of round 1 in the adaptive sampling strategy as an example to explain the mechanism behind the EI acquisition function. In Figure 8(b), compared to the widely distributed sampling positions in the initial sampling (round 0), the sampled positions in round 1 were concentrated at the low PC1 and high PC2 (top-left) region of the embedded space. The initial sampling used a clustering method based only on molecular information without any posterior, resulting in the selection of only the spatially representative molecules to be tested. In contrast, the round 1 sampling included the *R*_int_ calculated using CGMD on the molecules from the initial sampling and, therefore, preferred in sampling based on the trade-off between exploitation and exploration.

From Figure 8(c), the magnitudes of the exploitation term of all candidate molecules were obtained based on the *R*_int_ values either calculated or predicted by the GPR model. It is found that the favorable region with prominent exploitation terms is at the low PC1 *or* high PC2 (near the left and top edges of the data cluster). Similarly, in Figure 8(d), the magnitudes of the exploration terms are shown. In contrast, we noticed that the molecules with high exploration terms are at the low PC1 and high PC2 (top-left) regions, in agreement with the final sampled molecules in round 1, suggesting the dominant role of exploration in the sampling process in early rounds. Such dominance can be rationalized by revisiting eq 5, where the exploitation and exploration terms are additive. Since the region with high exploration values is a subset of the larger region with high exploitation values and the values of exploration are overall higher than exploitation based on the scaling bar comparison, the acquisition function in round 1 is dominated by exploration.

### 3.3 Result Analysis of High-Performing Candidates

#### 3.3.1 Bead Type Analysis of Surfactant Head

Figure 9 illustrates the probability distribution of bead types for the top 50 surfactants with the highest calculated interfacial resistance (*R*_int_ > 393 s/m), as identified by the active learning framework. This analysis aims to provide structural design principles for developing surfactant architectures with enhanced interfacial properties, rather than proposing specific chemical replacements. In the CG model, each surfactant is represented by a sequence of beads structured as C1–C1–C1–bead2–bead3–bead4–bead5, where bead5 terminates the head and bead2 links to the hydrocarbon tail.

**Figure 9:**
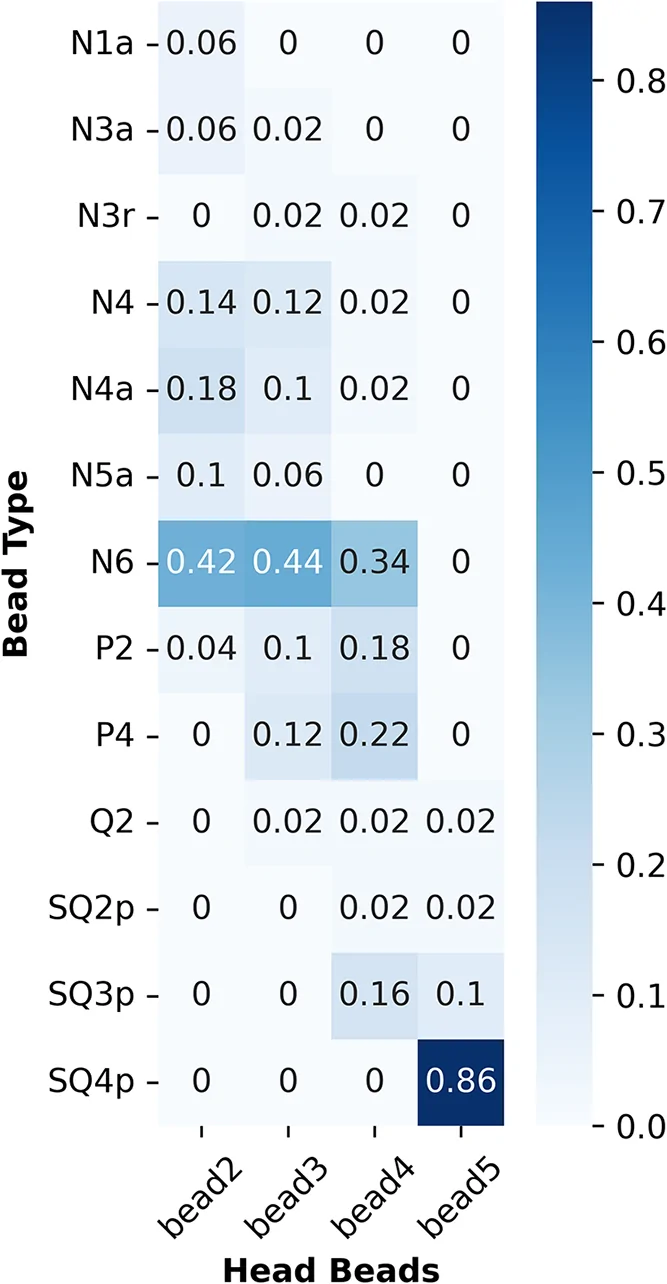
Probability distribution of bead types in the surfactant head for top 50 best-performing surfactants. Each surfactant features a CG structure of C1–C1–C1-bead2-bead3-bead4-bead5. The blue shading intensity reflects the magnitude of the probability, with larger numbers and darker shades representing higher probabilities.

Within this computational framework, the terminal position (bead5) appears predominantly occupied by charged ammonium-based groups (Q2, SQ2p, SQ3p, SQ4p). Interestingly, the occupancy probability of these groups correlates positively with their hydrophilicity, as quantified by oil–water transfer free energy and hydration energy:^27^ Q2 (tetramethylammonium) < SQ2p (alkyl dimethylammonium) < SQ3p (alkyl methylammonium) < SQ4p (alkyl ammonium). Notably, SQ4p, a charged alkyl ammonium group with a transfer free energy of −59.2 kJ/mol and hydration energy of 44.9 kJ/mol, exhibits a high occupancy probability of 0.86 at the terminal position. The simulations suggest that strong electrostatic interactions between these compact charged groups and the aqueous phase, combined with favorable self-interactions, may facilitate a robust barrier that impedes fuel transport across the surfactant monolayer. This observation suggests that the terminal headgroup plays a pivotal role in governing interfacial resistance, which is consistent with previous computational findings indicating that the narrow region between the surfactant head and the water phase primarily dictates transport kinetics.^18^

In contrast, the intermediate linker positions (bead2 to bead4) exhibit higher structural variability, suggesting that these beads influence performance but are less determinative than the terminal headgroup. Among these linker beads, N6, corresponding to a phenol-like aromatic group, frequently appears at connectable positions with a probability of approximately 0.4. The aromatic character of N6 may promote favorable aromatic or hydrophobic interactions between neighboring surfactants, thereby helping stabilize interfacial packing.^4^ In addition, the moderate polarity of N6 may facilitate a sharper transition between the hydrophobic tail region and the hydrophilic headgroup domain. These position-specific trends illustrate how the active learning pipeline can extract interpretable structure–property relationships from a large surfactant design space, providing molecular-level design guidance and a labeled data set for future experimental validation.

### 3.4 Comparative Analysis with Structural Analogs

The use of CG models facilitated a high-throughput exploration of the vast surfactant design space. To demonstrate the chemical relevance of the motifs identified by the active learning pipeline, we performed a back-mapping of the top-performing CG candidates (see Supporting Information) to representative all-atom structures. This step restores the atomistic detail simplified during the screening phase and provides a basis for evaluating the chemical plausibility of the proposed candidate structures. We subsequently compared these candidate structures with commercial surfactants to identify structural analogs.

For instance, one high-performing candidate is the aryl ether hydroxy ammonium amphiphile, denoted as AE-HA-1 (see Figure 10a), which is characterized by the bead sequence C1–C1–C1–N6–P4–P4–SQ4p. This architecture shares structural features with 1-amino-3-(4-octoxyphenoxy)­propan-2-ol (CAS No. 56704–21–3; see Figure 10b), a known surfactant capable of forming an −NH_3_^+^ group under specific conditions.^47^ Within the context of our workflow, the molecule represents a more complex variant of this structural motif, incorporating a longer alkyl chain and additional diol groups. According to our CGMD results, such structural modifications are associated with increased interfacial transport resistance; the C12 alkyl tail and additional hydrogen-bonding sites^48^ are expected to contribute to a more robust interfacial barrier.

**Figure 10:**
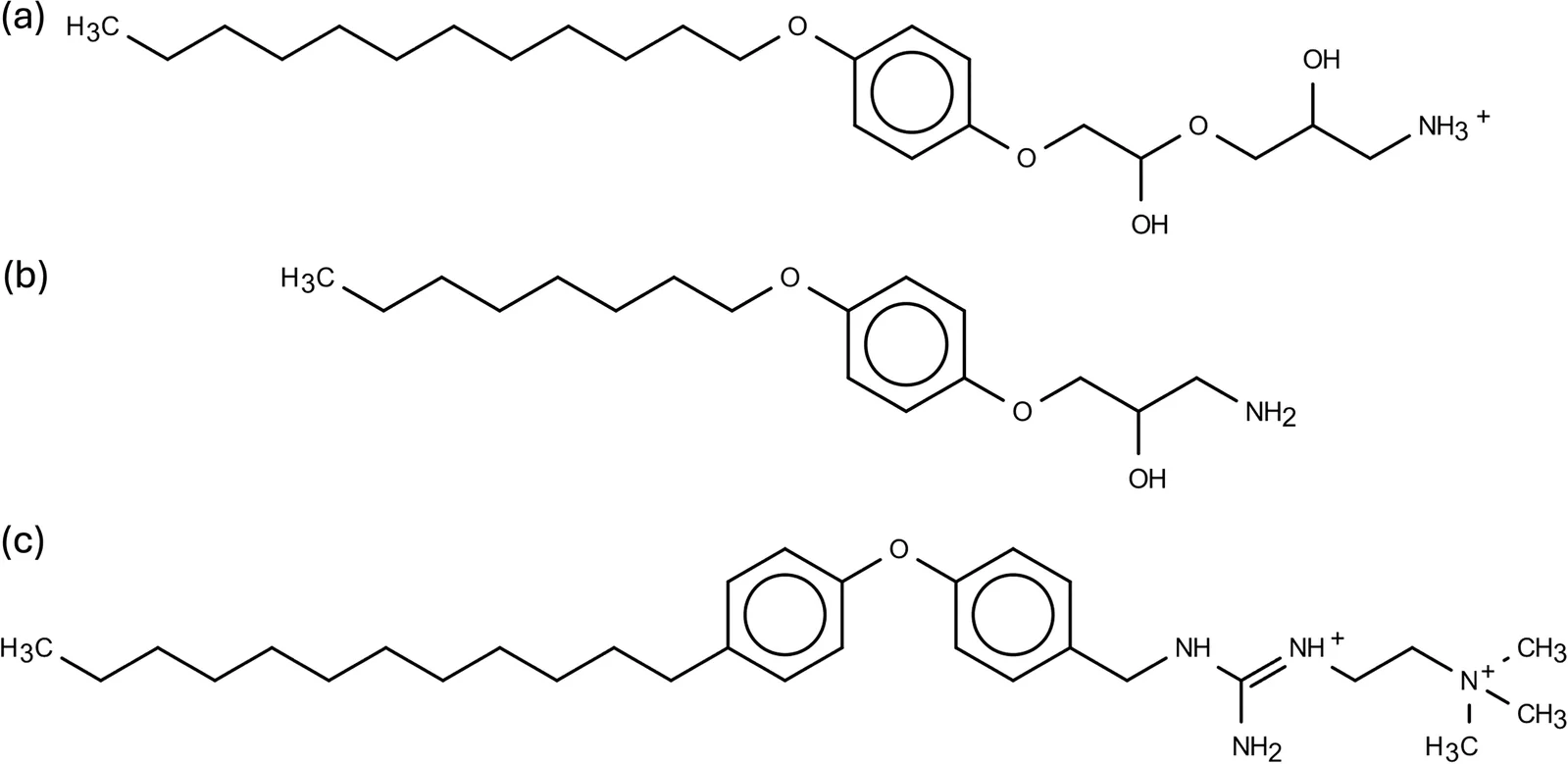
Chemical structures of our discovered top-performing surfactants and commercial surfactants. (a) AE-HA-1, an aryl ether hydroxy ammonium amphiphile. (b) 1-amino-3-(4-octoxyphenoxy)­propan-2-ol (CAS No. 56704–21–3). (c) DAE-GQ-1, a diaryl ether guanidinium-quaternary ammonium amphiphile. These 2D chemical structures were generated using the MolDraw Web site.^49^ Counterions were omitted for clarity.

Similarly, another candidate, DAE-GQ-1, a diaryl ether guanidinium-quaternary ammonium amphiphile, is represented by bead sequence C1–C1–C1–N6–N6–SQ3p–Q1 (see Figure 10c). Structurally, DAE-GQ-1 shares key features with commercial DOWFAX-type surfactants, including an alkyl-substituted diphenyl ether hydrophobic backbone. However, unlike traditional DOWFAX surfactants, which are anionic and typically contain two sulfonate groups, DAE-GQ-1 contains a guanidinium-quaternary ammonium dicationic headgroup instead. In this sense, it also resembles BAC-type cationic surfactants because of its permanently charged ammonium group and its expected affinity for the aqueous side of the interface. For PFAS-alternative design, this combined structure may be useful because the diphenyl ether backbone promotes strong interfacial residence and extreme thermochemical stability in the spirit of DOWFAX scaffolds,^50,51^ while the dicationic headgroup can enhance water-side anchoring and the persistence of the interfacial film.^52,53^ These possible advantages are based on the structural features of the molecule and should be viewed as a design hypothesis rather than an experimentally confirmed result.

Overall, this comparison demonstrates that the active learning pipeline can move beyond CG-level ranking to identify chemically plausible molecular motifs for future experimental evaluation. More broadly, the workflow provides a data-efficient strategy for exploring large PFAS-free surfactant design spaces without relying on exhaustive CGMD screening. By releasing the complete *R*_*int*_ data set and the associated open-source code, this work establishes a reusable computational resource for accelerating the synthesis, characterization, and optimization of PFAS-free surfactants for firefighting applications.

## 4 Conclusions

In summary, we have demonstrated a data-driven framework that combines active learning with CGMD simulations to efficiently explore the design space of hydrocarbon surfactants for inhibiting interfacial fuel transport. Our study focused on a representative library of single-chain surfactants with 12-carbon hydrocarbon tails. Using transferable CG models together with multistage screening for stability and synthetic feasibility, we reduced an initial space of approximately 2.8 million candidates to a targeted library of 12,124 unique CG surfactants. These surfactants were represented by latent vectors learned from molecular graphs using a GNN-based autoencoder, and a GPR surrogate model combined with Bayesian Optimization was used to guide candidate selection during the discovery process. Within this computational framework, we identified high-performing candidates with interfacial transport resistance (*R*_*int*_) reaching 1641.5 s/m, representing a significant improvement over the commercial DTAB benchmark.

Position-specific bead analysis of the top 50 candidates suggests that the terminal head plays a decisive role in governing transport resistance, likely because of strong electrostatic interactions, whereas the linker positions appear to have a less determinative effect on performance. This work highlights the power of active learning to transform PFAS-free surfactant discovery from an exhaustive screening problem into a data-efficient optimization task. By strategically selecting candidates for CGMD evaluation, the framework efficiently navigates a large chemical space and identifies high-*R*_*int*_ surfactants while avoiding the prohibitive cost of simulating every candidate. The resulting workflow provides a scalable computational strategy for accelerating the design and experimental prioritization of PFAS-free surfactants for firefighting applications. To support future research and experimental validation, the complete *R*_int_ data set for all sampled surfactants and the active learning code are publicly available in our GitHub repository at https://github.com/elliewang21/Active_Learning.

## Supplementary Material



## Data Availability

The data set of interfacial transport resistance values for the 331 surfactants from both sampling strategies, along with the code for our active learning workflow, is available at https://github.com/elliewang21/Active_Learning.

## Notes

Molecular graph representations, autoencoder architecture and training procedure, hyperparameters of the regularized autoencoder (RAE) and Gaussian process regression (GPR) models, and the interfacial transport resistance (*R*_int_) results of the top 10 CG surfactants (PDF)
