# Supplementary material for: An Active Learning Framework for PFAS-Free Surfactant Discovery in Firefighting Foams
Source: ACS Omega. 2026 Sep 30;11(40):61846–57. doi: 10.1021/acsomega.6c07702 (PMC13670300; doi:10.1021/acsomega.6c07702)
Supplement: Supplementary file 1 [file ao6c07702_si_001.pdf]

Supplementary Information

An Active Learning Framework for PFAS-Free Surfactant Discovery in Firefighting Foams

Xin Wang[a,†,*], Hongwei Zhang[a,†], Xingtong Yu[b], Seokgyun Ham[a], Brian Lattimer[a], Rui Qiao[a,*]

[a]Virginia Tech, Department of Mechanical Engineering, 635 Prices Fork Road, Blacksburg, VA 24061, United States

[b]The Chinese University of Hong Kong, Department of Systems Engineering and Engineering Management, Ma Liu Shui, New Territories, 999077, Hong Kong, China

[†]These authors contributed equally to this work.

[*]Corresponding authors: xinwang@vt.edu; ruiqiao@vt.edu.

1 Chemical space of CG surfactants

Surfactant head groups were constructed from Martini 3 bead library [1]. The head-group library contained 28 unique bead types, including 11 terminal-only beads and 17 normal beads, as summarized in Table S1. Terminal-only beads were allowed only at the terminal position of the surfactant head group, whereas normal beads were allowed at both internal/connectable positions and terminal positions. Thus, the normal bead pool was a subset of the full bead pool, which contained both terminal-only and normal beads. Head groups containing one to four beads were generated sequentially from the hydrocarbon tail toward the terminal head position. Internal positions were selected from the normal bead pool, and the terminal position was selected from the full bead pool.

Table S1: 28 unique Martini 3 beads used to construct surfactant head groups. The "terminal-only" beads were allowed only at the terminal position of the head group, whereas "normal" beads were allowed at both internal/connectable positions and terminal positions. The bead charge and oil-to-water transfer free energies, $\Delta G_{\text{oil}\rightarrow\text{water}}$, were obtained from the Martini 3 force field [1].

**Bead**	**Charge**	$\Delta G_{\text{oil}\rightarrow\text{water}}$ **(kJ mol^{-1})**	**Role**
SQ4p	+1	-59.2	terminal-only
Q5n	-1	-64.1	terminal-only
Q4n	-1	-48.3	terminal-only
D	-2	-78.8	terminal-only
N2ah	0	0.5	terminal-only
N2a	0	0.5	terminal-only
N6d	0	-9.8	terminal-only
P1	0	-11.1	terminal-only
P5	0	-19.2	terminal-only
P3	0	-15.0	terminal-only
P3a	0	-15.0	terminal-only
N2	0	0.5	normal
N5a	0	-7.5	normal
P6	0	-27.2	normal
P2	0	-13.2	normal
Q1	+1	-24.2	normal
SQ3p	+1	-47.8	normal
SQ2p	+1	-43.3	normal
Q2	+1	-32.7	normal
Q5	-1	-64.1	normal
N1a	0	2.9	normal
N3r	0	-3.6	normal
N4a	0	-5.0	normal
N4	0	-5.0	normal
N3a	0	-3.6	normal
P4	0	-17.1	normal
N1	0	2.9	normal
N6	0	-9.8	normal

2 Molecular graph representations

The molecular representation of candidate molecules is shown in Table S2 in the form of molecular graphs. The node representations consist of both numerical and categorical features. Node features describe the physicochemical properties of individual beads, including charge, oil-to-water transfer free energy, bead type, hydrogen-bonding character, charge label, self-interaction label, and bead size. Edge features encode the Lennard-Jones interaction parameters, σ and ϵ,

between connected beads.

Table S2: Molecular representations of molecules in the chemical space.

Parameter	Description	Value
	**Node features**	
q_{bead}	The charge on bead	-2, -1, 0, +1
q_{tot}	The total charge of molecule	[-4, +4]
ΔG	The oil to water free energy	ref. Martini3[1]
Categorical	Bead type	N1, N2, N3, N4, N5, N6, P1, P2, P3, P4, P5, P6, Q1, Q2, Q3, Q4, Q5, D, C1
Categorical	Hydrogen bonding label	d/a
Categorical	Positive and negative label	p/n
Categorical	Self-interaction label	h/r
Categorical	Bead size label	S/R
	**Edge features**	
σ	The distance where inter-bead potential is zero	ref. Martini3[1]
ϵ	The potential well depth	ref. Martini3[1]

3 Autoencoder architecture and training protocol

An autoencoder consists of an encoder and a decoder. The encoder takes in the graph representations of molecules as inputs where all the categorical node features are represented using one-hot encoding. Structure-wise, the encoder first uses a linear embedding layer to embed the one-hot bead type description to a 32-dimensional vector. This vector is then concatenated to the rest of the node features and passed into another linear embedding layer to form a new 64-dimensional node vector. The new node vector is passed into a gated Message Passing Neural Network (MPNN).[2] In MPNN, the two edge features are mapped into a 64×64 matrix. Five rounds of message passing were performed, and the hidden states were aggregated to the 16-dimensional embedded vector using the set2set model[3] and a Multilayer perceptron (MLP).

The decoder here is designed to reconstruct the original graph representations based solely on the embedded 16-dimensional vectors. This ensures that the embedded vectors have learned enough information to be used for the later active learning sampling process. In the decoder, the embedded vectors are first passed to 8 different MLPs to reproduce the 8 node features in Table S2, separately. To reconstruct the adjacency matrix, the reproduced node feature vectors of any

combination of two nodes are then passed separately to an MLP to extract the information. The extracted two vectors are averaged (the adjacency information is invariant to the order of two nodes) and concatenated to the output of another MLP that takes in the 16-dimensional embedded vector as input. This concatenated vector is then passed to a third MLP to reproduce a specific adjacency matrix entry. The edge feature matrices are not reconstructed because the edge features are directly related to the nodes they are connected to.

Table S3: Hyperparameters of the regularized autoencoder (RAE) and Gaussian process regression (GPR) models.

Model	Hyperparameter	Value
RAE	Bead type embedding dimension d_e	32
	Hidden state dimension d_h	64
	Message passing steps T	5
	Latent space dimension d	16
	Batch size	64
	Learning rate	3.5×10^{-4}
	RAE regularization weight λ	2.0
	Maximum number of epochs	5000
GPR	Kernel	Linear + RBF
	Label transformation	Yeo-Johnson
	Optimizer	Adam
	Learning rate	1×10^{-2}
	Maximum number of epochs	5000

During the RAE training, the loss function is the summation of the reconstruction loss and the regularization loss, which are shown in Equations (S1)–(S4). All the hyperparameters are summarized in Table S3.

$$\mathcal{L} = \mathcal{L}_{\mathrm{REC}} + \lambda \mathcal{L}_{\mathrm{RAE}} \tag{S1}$$

$$\mathcal{L}_{\mathrm{REC}} = \min_{\pi \in \mathcal{S}_N} \left[\sum_k \mathrm{BCE}\Big(\hat{N}_\pi^k,\, N^k\Big) + \mathrm{BCE}\Big(\hat{A}_\pi,\, A\Big) \right] \tag{S2}$$

$$\mathcal{L}_{\mathrm{RAE}} = \frac{1}{2} \|z\|_2^2 \tag{S3}$$

where

$$\text{BCE}(P, Y) = -\frac{1}{|P|} \sum_i \left[Y_i \log P_i + (1 - Y_i) \log(1 - P_i)\right] \tag{S4}$$

Here, $k \in \{\text{NM}, \text{HB}, \text{PN}, \text{SI}, \text{SZ}\}$ indexes the five categorical node feature groups, corresponding to bead type, hydrogen-bonding label, positive and negative label, self-interaction label, and bead size label, respectively. Here, $\pi \in \mathcal{S}_N$ denotes a permutation of the N beads, and $\mathcal{S}_N$ is the set of all such permutations. In this work, $N = 5$, so $|\mathcal{S}_N| = N! = 120$, and $\lambda = 2.0$.

4 Interfacial transport resistance (R_{int}) results of top 10 CG surfactants

After 4 rounds of active learning and testing 300 candidates for each BO strategy, the results of top 10 CG surfactants are summarized in Table S4. The source code and all R_{int} data collected from the CGMD simulations are publicly available on GitHub: `https://github.com/elliewang21/Active_Learning`.

Table S4: R_{int} results of top 10 CG surfactants

**Index**	**Bead Representations**	R_{int} **(s/m)**
10441	C1-C1-C1-N6-P4-P4-SQ4p	1641.5
10655	C1-C1-C1-N6-N6-SQ3p-SQ3p	1175.7
6283	C1-C1-C1-N4-N6-P4-SQ4p	967.6
4407	C1-C1-C1-N4a-N6-SQ3p-Q2	900.7
10656	C1-C1-C1-N6-N6-SQ3p-SQ2p	793.9
2249	C1-C1-C1-N1a-N6-P2-SQ4p	782.3
10245	C1-C1-C1-N6-P2-P2-SQ4p	746.0
9071	C1-C1-C1-N6-N3r-SQ3p-SQ4p	699.7
5266	C1-C1-C1-N5a-P4-N6-SQ4p	691.2
5475	C1-C1-C1-N5a-N6-P4-SQ4p	620.5

References

(1) Souza, P. C.; Alessandri, R.; Barnoud, J.; Thallmair, S.; Faustino, I.; Grünewald, F.; Patmanidis, I.; Abdizadeh, H.; Bruininks, B. M.; Wassenaar, T. A., et al. Martini 3: a general purpose force field for coarse-grained molecular dynamics. *Nature methods* **2021**, *18*, 382–388.

(2) Gilmer, J.; Schoenholz, S. S.; Riley, P. F.; Vinyals, O.; Dahl, G. E. In *International conference on machine learning*, 2017, pp 1263–1272.

(3) Vinyals, O.; Bengio, S.; Kudlur, M. Order matters: Sequence to sequence for sets. *arXiv preprint arXiv:1511.06391* **2015**.
